# Determining the existence of the foramen of Huschke in patients with temporomandibular joint disorders using cone beam computed tomography: retrospective cohort study

**DOI:** 10.1186/s12880-022-00850-1

**Published:** 2022-08-13

**Authors:** Mahvash Hasani, Abdolaziz Haghnegahdar, Leila Khojastepour, Mohammad Javad Golbahar Haghighi

**Affiliations:** 1 Department of Oral and Maxillofacial Radiology, School of Dentistry, Shiraz University of Medical Science, Shiraz, Iran; 2 School of Dentistry, Shiraz University of Medical Science, Shiraz, Iran

**Keywords:** Foramen of Huschke, External auditory canal, Temporomandibular disorders, Cone beam computed tomography

## Abstract

**Background:**

Foramen of Huschke has been presented as an unusual developmental defect in anteroinferior aspect of external auditory canal. It can be associated with significant otologic complications. The purpose of this study was to determine the association between existence of foramen of Huschke and temporomandibular joint disorders in Cone Beam Computed Tomography (CBCT) images.

**Methods:**

Of an initial sample of 465 patients, we retrospectively evaluated the CBCT images of 118 individuals with clinical signs and symptoms of temporomandibular joint disorders as case group and 256 individuals as control group. The presence, size and localization of foramen of Huschke were assessed in the axial and corrected sagittal images. The sex and age distribution were determined. Fisher’s exact test, T-test and Pearson’s Chi-square were applied to assess the relationship between foramen of Huschke and temporomandibular joint disorders in the case and control groups considering age and sex.

**Results:**

The foramen of Huschke prevalence was slightly higher in patients with temporomandibular joint disorders (3.4%) than patients without temporomandibular joint disorders (0.8%). However, the difference was not statistically significant (P = 0.082). foramen of Huschke was found in five females and one male. There was no significant difference between case and control groups considering the age of patients with foramen of Huschke (P = 0.683). There was no significant difference between the case and control groups, considering the right and left ears in distribution of foramen of Huschke (P = 0.099) (P = 0.183).

**Conclusions:**

Higher prevalence of foramen of Huschke in patients with temporomandibular joint disorders may suggest possible mechanism for temporomandibular joint disorders development that can be affected by presence of foramen of Huschke.

## Background

The foramen of Huschke (FH) is an anatomical variation of the anteroinferior portion of the external auditory canal (EAC) which is located posteromedial to the temporomandibular joint (TMJ) [1, 2]. This structure is involved in embryonic development of the tympanic portion of the temporal bone and serves as an anatomical separator between EAC and TMJ. The EAC closure commonly occurs at 5 years of age, whereas FH may not be closed after this age [3]. FH is also described as an abnormal structure that developed due to defective ossification of the tympanic bone and joining the anterior and posterior prominences of the tympanic ring [4].

In 1987, the first case of the TMJ herniation into the EAC through a patent foramen of Huschke was described [5]. Trauma, inflammation, and a mass effect are well-known causes of herniation of the TMJ into the EAC [1, 3, 4]. Spontaneous herniation of the TMJ soft tissue into the EAC through the foramen of Huschke is extremely rare, with approximately 30 cases reported in the literature [1, 6–8]. The symptoms of FH include conductive hearing loss, otalgia, otorrhea, and clicking tinnitus; although it may be asymptomatic [5–7].

Preoperative detection of the foramen of Huschke is of high importance for clinicians, especially those who perform ear and TMJ surgery for patient suffer TMJ disorders. During TMJ arthroscopy, endoscopes smaller than 3 mm in diameter may cause tympanic membrane perforation, incus dislocation, and facial nerve tympanic segment damage by penetrating into the foramen [9]. So far, few studies have focused on synchronization between TMJ disorders and the presence of FH [1, 5–9]. Nevertheless, the literature lacks studies about presence of FH in patients with TMJ disorders, and the relationship between this abnormality and the TMD is unclear. So, awareness about the presence of FH in TMD can be of high significance.

Recent guidelines recommend Cone-beam computed tomography (CBCT) as the modality of choice for evaluation of temporomandibular joint osseous changes because of lower radiation dose, higher spatial resolution and the growing availability of CBCT than conventional CT [10–12]. Due to the superimposition of temporal bone structures, including the tympanic portion, identification of FH is very challenging or even impossible by conventional methods, such as conventional linear tomography and panoramic radiography. CBCT has been successful in identifying FH [13–15].

Given this background, this study aimed to evaluate the association between the presence of FH and TMD and to compare the presence of FH in patients with and without TMD on CBCT images. The null hypothesis in this research was that patients with TMD presented same prevalence of FH as the control group.

## Methods

 This case-control study was approved by the ethics committee of Shiraz University of Medical Sciences, Shiraz, Iran (#IR.SUMS.DENTAl.REC.1399.064). This study was in compliance with the Helsinki Declaration. Informed contest was taken from all patients or their guardians at the time of the radiographic examination. Patients^,^ data were extracted from their records from the archive of the Department of Maxillofacial Radiology at Shiraz Dental School. In our department, all TMD patients were examined by maxillofacial postgraduate students under the supervision of maxillofacial radiologists with at least 8 years’ experience. Clinical signs and symptoms that were examined were joint pain, muscle pain, mouth-opening limitation, joint noise (click or crepitation), and nonharmonic movements of the joint. CBCT was required for those patients who were suspected of osseous deformities of the jaws such as degenerative joint disease.

Of an initial sample of 465 patients, referred to our department between June 2018 and June 2020, a total of 118 patients with TMD were recruited as a case group and 256 patients without TMD were considered as a control group. Patients with a history of inflammation, cholesteatoma, and trauma or surgery in the TMJ region, as well as those under 5 years of age, were not included in this study.

The CBCT images were obtained, using a flat panel detector (FPD) scanner (New Tom VGi, QR s.r.l., Italy). All images were acquired within a total exposure time of 1.8 s at 110 kVp, using a field of view of at least 15 × 12 cm^2^; the electrical current (mA) was adjusted automatically for each patient. All CBCT images were obtained in a standardized head posture (the Frankfurt plane parallel to the floor). The CBCT images were reviewed in NNT viewer version 8.0. CBCT images were viewed and reported in a dimly lit, quiet room, using a medical grade diagnostic display device (Barco monitor, Model: MDRS-2122, Belgium). Image adjustments such as zoom, brightness, and contrast were also permitted. The CBCT images were analyzed by two observers, one 6th year dental student and one maxillofacial radiologist with 8 years experience. The level of intra- and inter-examiner agreement was assessed.

**Fig. 1:**
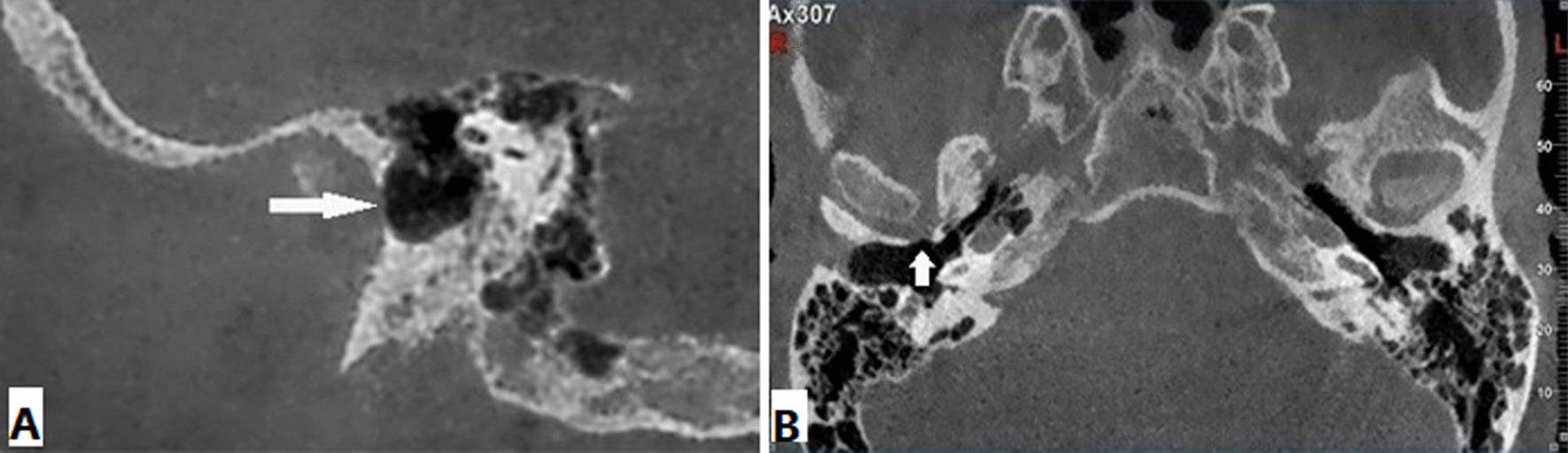
FH (white arrow) in the sagittal image (**A**), in the axial image (**B**)

**Fig. 2:**
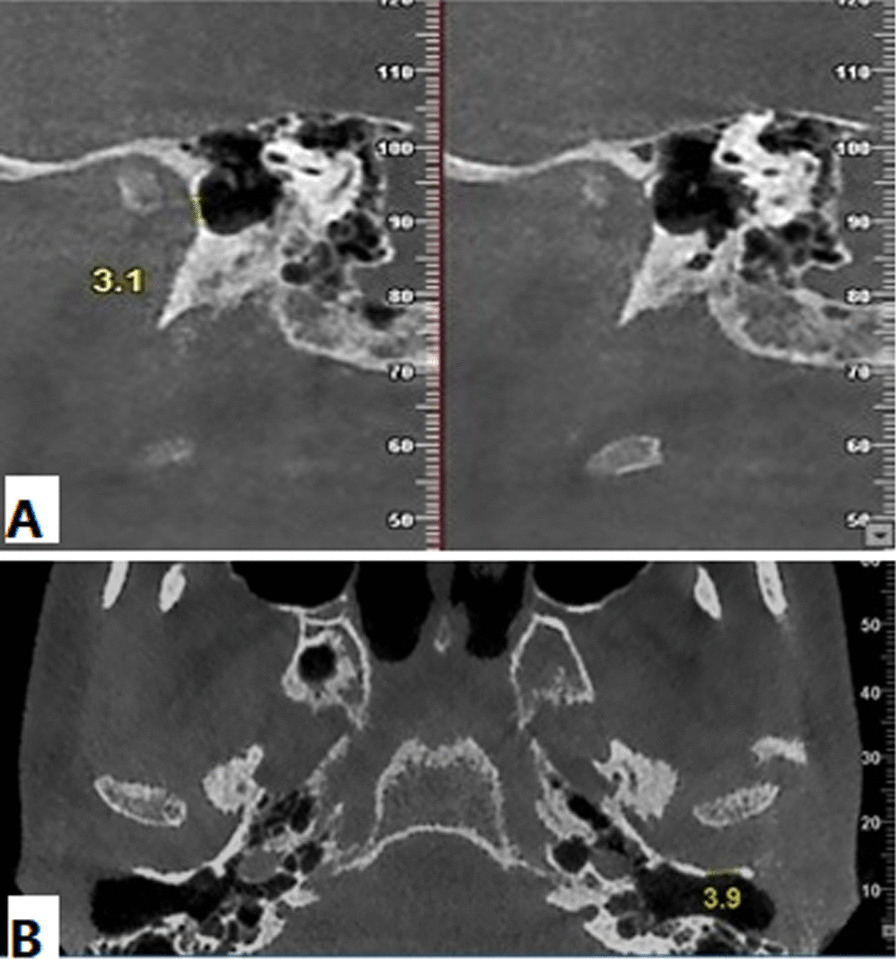
The dimension of FH in the sagittal image (**A**) and axial image (**B**)

The anteroinferior aspect of EAC were assessed at a slice thickness of 1 mm and a slice interval of 1 mm to assess the presence of FH. The presence of FH was evaluated in the axial plane. The corrected sagittal images were examined for verification (Fig. 1). In patients with FH, we measured the size of the foramen in the axial and reformatted sagittal planes in millimeter (Fig. 2). Moreover, we determined the FH localization (left and right), age, and sex distribution.

### Statistical analysis

Statistical analysis was performed using SPSS version 26.0 (SPSS Inc., Chicago, IL, USA). The level of significance was set at *P* < 0.05. Fisher’s exact test was applied to assess the relationship between FH and TMD in the case and control groups. For descriptive statistics, frequency, percentage, mean, standard deviation (SD), minimum, and maximum values were determined in both groups. T-test was used to compare the age distribution with presence of FH in the case and control groups. Moreover, Pearson’s Chi-square test was exploited to compare the sex distribution between the groups.

## Results

There was an almost perfect intra-observer and inter-observer agreement (ICC = 0.87, 0.89 respectively). A total of 55 male and 63 female patients, with the mean age of 35.43 years (SD = 15.11), were included in the case group, while 123 male and 133 female patients, with the mean age of 33.24 years (SD = 14.06), were included in the control group. The minimum age of the control and case subjects was 7 and 11 years, respectively, while their maximum age was 74 and 73 years, respectively. The patients’ age and sex distributions are presented in Table 1. There was no significant difference considering sex (*P* = 0.824) or age (*P* = 0.172) between the case and control groups.

**Table 1:** Sex and age distribution of patients with and without FH

Groups	Sex		Mean age
	Female N (%)	Male N (%)	Years (± SD)
Case	63 (53.4)	55 (46.6)	35.43 (± 15.11)
Control	133 (52.0)	123 (48.0)	33.24 (± 14.06)
Total	196 (52.4)	178 (47.6)	33.93 (± 14.41)

Overall, we found FH in 6 (1.6%) out of 374 patients. FH was present in 4 (3.4%) cases and 2 (0.8%) controls. Of six patients with FH, one patient showed bilateral FH, and five patients showed unilateral FH. FH was located on the left side in 4 (1.1%) patients and on the right side in 2 (0.5%) patients (Table 2). The shape of the foramen was considered oval, as its dimensions were slightly different in the two planes. The mean size of the foramen was 3.28 mm (range: 2.7–4.8 mm) in the axial plane and 2.94 mm (range: 2.6–3.4 mm) in the sagittal plane. There was no significant difference between the case and control groups, considering the right (*P* = 0.099) and left (*P* = 0.183) ears.

**Table 2:** Laterality and size distribution of patients with and without FH

Groups	Laterality		Mean Size	
	Left N	Right N	Axial (mm)	Sagittal (mm)
Case	3	2	3.28	2.94
Control	2	0	4.7	3
Total	5	2	3.68	2.95

The prevalence of FH in patients with TMD (3.4%) was slightly higher than that of subjects without TMD (0.8%); however, the difference was not significant (*P* = 0.082). FH was found in five females and one male. The mean age of patients with FH was 38.66 years (SD = 21.51), there was no significant difference between case and control groups considering the age of patients with FH (P = 0.683).

## Discussion

FH is a developmental defect, positioned posteromedial to TMJ in the tympanic portion of the temporal bone. It is normally closed during growth before 5 years of age. However, it may not close in some cases and may remain present during life. The prevalence of FH ranges from 1.5 to 43% in different studies [3, 7, 8, 16]. Factors, such as inflammation, tumor, and trauma, may lead to TMJ protrusion into the EAC. Also, FH may be associated with herniation of soft tissues from TMJ to EAC and may predispose individuals to TMD [17–20]. Mandibular pressure on the tympanic bone during activities, such as deglutition, mastication, and respiration may result in the lack of FH closure [1, 21].

Presence of FH may be asymptomatic, and therefore, patients with this variation may be overlooked. The signs and symptoms of FH include middle ear inflammation, tinnitus, hearing loss, otalgia, and TMJ pain [22, 23]. Few studies have reported TMJ herniation through FH into the EAC. However, they only reported this finding and did not describe any relationship between TMD and FH [9, 24–29]. The present study is the first to evaluate the relationship between TMD and the presence of FH.

CBCT delivers a lower dose of radiation than conventional CT for imaging anatomical landmarks in the maxillofacial region. The quality of CBCT images of maxillofacial structures is comparable to that of conventional CT [19, 20]. However, CBCT is distinct from conventional CT, as complete data can be obtained during a single scan. Also, the exposure phase is usually less than 20 s. Although soft tissue resolution of CBCT is inferior to medical CT, CBCT can produce images of bony structures with higher resolution and lower amount of radiation [23].

Some studies have used CBCT to investigate the prevalence and characteristics of FH with different slice thicknesses and exposure conditions in healthy populations and people with disorders. In cadaveric studies, the prevalence of FH ranged from 4 to 43% [30, 31]. Although in radiological studies, the prevalence of FH is between 1.5 and 23% [7, 32], according to a meta-analysis by Pekala et al. [33] FH was more prevalent in cadaveric studies (21.2%) than radiological studies (8.8%). In the present study, the prevalence of FH was 1.6%; it was bilateral in one patient and unilateral in five patients. In line with our study, Park et al. found that the prevalence of FH was 1.5% in all examined cases [7].

Moreover, in previous studies, the shape of the foramen was found to be oval, owing to different sizes of the foramen in axial and sagittal images. In agreement with studies by Hasani et al. and Lacout et al. [1, 16, 17] we found that the shape of FH is oval due to slightly different dimensions in the two planes.

The causes and primary pathophysiology of TMD have been controversial subjects over the years. Researchers mentioned possible association between occlusal factors (grinding and clenching), psychological factors (depression and anxiety), hormonal factors (estrogen receptors in female’s TMJ), trauma, and presence of the FH [12, 34, 35]. The signs and symptoms of TMD include pain in TMJ or mastication muscles which may radiate to local and distant structures, popping, clicking, or crepitus of TMJ on movement, with or without locking the joint; and headache and otalgia or tinnitus (or both) in the absence of aural disease [11, 12, 34]. Choi et al. and Lim et al. reported cases of clicking tinnitus with clenching teeth, while there was no history of trauma or surgery in the TMJ region. The CT scan of the temporal bone indicated a bony defect in the posteromedial wall of glenoid fossa, and the otoscopic examination indicated TMJ herniation through FH into EAC [28, 36].

Till now researches have focused on synchronization between temporomandibular joint herniation and the presence of FH. Preoperative detection of FH in patients with TMD has clinical significance and can affects treatment plan for these patients.

According to the present study, the prevalence of FH was 3.4% in subjects with TMD and 0.8% in subjects without TMD. The data analysis indicated that the prevalence of FH was slightly higher in subjects with TMD, compared to those without it; however, the difference was not statistically significant (*P* = 0.082). From six patients with FH, five patients showed unilateral FH and one patient showed bilateral FH. FH was located on the left side in four (1.1%) patients and on the right side in two (0.5%) patients. FH was found in five females and one male. The mean age of patients with FH was 38.66 years (SD = 21.51), considering the age of patients with FH there was no significant difference between case and control groups (P = 0.683). Regarding *P* value of our study (0.082), we suggest higher sample sizes to scrutinize this relationship more reliably.

Overall, we compare the presence of FH in subjects with and without TMD. Due to the limitations of CBCT data collection, we recommend similar studies with a larger sample size.

## Conclusions

Although prevalence of FH was higher in subjects with TMD than in subjects without TMD. However, there was no significant difference in the prevalence of FH between the case and control groups. The results of this study should to be validated with larger sample sizes in future studies.

## Data Availability

The data that support the findings of this study are available from Shiraz University of Medical Sciences but restrictions apply to the availability of these data, which were used under license for the current study, and so are not publicly available. Data are however available from the authors upon reasonable request and with permission of Shiraz University of Medical Science.

## References

[1] Lacout A, Marsot-Dupuch K, Smoker WR, Lasjaunias P (2005). Foramen tympanicum, or foramen of Huschke: pathologic cases and anatomic CT study. AJNR Am J Neuroradiol.

[2] Duman ŞB, Yeşiltepe S, Bayrakdar İŞ, Yaşa Y-s (2020). Retrospective comparison of cleft lip/palate patients and normal controls: cone beam computed tomograph imaging of foramen Husckhe morphology. Eur J Anat.

[3] Wang R-G, Bingham B, Hawke M, Kwok P, Li J (1991). Persistence of the foramen of Huschke in the adult: an osteological study. J Otolaryngol.

[4] Fusconi M, Benfari G, Franco M, Deriu D, Dambrosio F, Greco A (2009). Foramen of Huschke: case report and experimental procedure for diagnosis of spontaneous salivary fistula. J Oral Maxillofac Surg.

[5] Hawke M, Kwok P, Mehta M, Wang R-G (1987). Bilateral spontaneous temporomandibular joint herniation into the external auditory canal. J Otolaryngol.

[6] Moriyama M, Kodama S, Suzuki M (2005). Spontaneous temporomandibular joint herniation into the external auditory canal: a case report and review of the literature. Laryngoscope.

[7] Park YH, Kim HJ, Park MH (2010). Temporomandibular joint herniation into the external auditory canal. Laryngoscope.

[8] Kim TH, Lee SK, Kim SJ, Byun JY (2013). A case of spontaneous temporomandibular joint herniation into the external auditory canal with clicking sound. Korean J Audiol.

[9] Ertugrul Suha MD (2018). Rare cause of tinnitus: spontaneous temporomandibular joint herniation into the external auditory canal. J Craniofac Surg.

[10] Barghan S, Tetradis S, Mallya S (2012). Application of cone beam computed tomography for assessment of the temporomandibular joints. Aust Dent J.

[11] Murphy MK, MacBarb RF, Wong ME, Athanasiou KA (2013). Temporomandibular joint disorders: a review of etiology, clinical management, and tissue engineering strategies. Int J Oral Maxillofac Implants.

[12] Gauer R, Semidey MJ (2015). Diagnosis and treatment of temporomandibular disorders. Am Fam Physician.

[13] Tozoğlu U, Caglayan F, Harorlı A (2012). Foramen tympanicum or foramen of Huschke: anatomical cone beam CT study. Dentomaxillofac Radiol.

[14] Deniz Y, Geduk G, Zengin A (2018). Examination of foramen tympanicum: an anatomical study using cone-beam computed tomography. Folia Morphol.

[15] Tucunduva RMA, Lopes IA, Shinohara AL, Lauris JRP, Rubira-Bullen IRF (2019). Usefulness of cone-bean computed tomography exams to detect foramen of huschke in diverse age group. J Craniofac Surg.

[16] Hasani M, Shahidi S, Hasani M, Pourhoseini AH (2018). Cone beam CT study of the foramen of huschke: prevalence, and characteristics. Curr Med Imaging Rev.

[17] Weissman JL, Hirsch BE, Chan K, Tabor EK, Curtin HD (1991). Dehiscent temporomandibular joint. Radiology.

[18] Çakur B, Sümbüllü MA, Durna D, Akgül HM (2011). Prevalence of the types of the petrotympanic fissure in the temporomandibular joint dysfunction. Acta radiol.

[19] Langer J, Begall K (2004). Otosialorrhoea-diagnostics and therapy of a salivary fistula of the external auditory canal. Laryngorhinootologie.

[20] Tasar M, Yetiser S, Saglam M, Tasar A (2003). Congenital common cavity deformity of frontal, ethmoid, and sphenoid sinuses. Cleft Palate Craniofac J.

[21] Mao JJ, Nah H-D (2004). Growth and development: hereditary and mechanical modulations. Am J Orthod Dentofacial Orthop.

[22] Toyama C, da Silva C, Fugita D, Scapini F (2009). Temporomandibular joint herniation into the external auditory canal. Otol Neurotol.

[23] Rushton V, Pemberton M (2005). Salivary otorrhoea: a case report and a review of the literature. Dentomaxillofac Radiol.

[24] Mittal S, Singal S, Mittal A, Singal R, Jindal G. Identification of foramen of Huschke with reversible herniation of temporomandibular joint soft tissue into the external auditory canal on multidetector computed tomography. Taylor & Francis; 2017:92–3. 10.1080/08998280.2017.11929544 PMC5242129 28127148

[25] Ryu KH, Baek HJ, Hur DG (2017). Spontaneous temporomandibular joint herniation into the external auditory canal through a patent foramen of Huschke: a case report. Ann Med Surg.

[26] Williams RA, Jackler RK, Corrales CE (2017). Benign temporomandibular joint lesions presenting as masses in the external auditory canal. Otol Neurotol.

[27] Burlak K, So TY, Maclaurin WA, Dixon AF (2018). Foramen tympanicum with symptomatic temporomandibular joint herniation. Radiol Case Rep.

[28] Lim K, Jung J, Rhee J, Choi J (2019). Temporomandibular joint herniation through the foramen of Huschke with clicking tinnitus. Eur Ann Otorhinolaryngol Head Neck Dis.

[29] Kinar A, Bucak A, Ulu Ş (2020). An otalgia cause: temporomandibular joint herniation from foramen of huschke to external auditory canal. J Craniofac Surg.

[30] Rezaian J, Namavar MR, Nasab HV, Nobari ARH, Abedollahi A (2015). Foramen tympanicum or foramen of huschke: a bioarchaeological study on human skeletons from an iron age cemetery at tabriz kabud mosque zone. Iran J Med Sci.

[31] Bhanu PS, Sankar KD (2016). Incidence of foramen of Huschke in South Andhra population of India. Clin Diagn Res.

[32] Akbulut N, Kursun S, Aksoy S, Kurt H, Orhan K (2014). Evaluation of foramen tympanicum using cone-beam computed tomography in orthodontic malocclusions. J Craniofac Surg.

[33] Pękala JR, Pękala PA, Satapathy B, Henry BM, Skinningsrud B, Paziewski M (2018). Incidence of foramen Tympanicum (of Huschke): comparing cadaveric and radiologic studies. J Craniofac Surg.

[34] Scrivani SJ, Keith DA, Kaban LB (2008). Temporomandibular disorders. N Engl J Med.

[35] Chisnoiu AM, Picos AM, Popa S, Chisnoiu PD, Lascu L, Picos A (2015). Factors involved in the etiology of temporomandibular disorders-a literature review. Clujul Med.

[36] Choi JW, Nahm H, Shin JE, Kim C-H (2020). Temporomandibular joint herniation into the middle ear: a rare cause of mastication-induced tinnitus. Radiol Case Rep.

